# Tattoos and Risk of Hematologic Cancer: A Population‐Based Case–Control Study in Utah

**DOI:** 10.1002/cam4.70260

**Published:** 2024-10-23

**Authors:** Rachel D. McCarty, Britton Trabert, David Kriebel, Morgan M. Millar, Brenda M. Birmann, Laurie Grieshober, Mollie E. Barnard, Lindsay J. Collin, Katherine A. Lawson‐Michod, Brody Gibson, Jenna Sawatzki, Marjorie Carter, Valerie Yoder, Jeffrey A. Gilreath, Paul J. Shami, Jennifer A. Doherty

**Affiliations:** ^1^ Huntsman Cancer Institute, University of Utah Salt Lake City Utah USA; ^2^ Department of Population Health Sciences Spencer Fox Eccles School of Medicine University of Utah Intermountain Healthcare, University of Utah Salt Lake City Utah USA; ^3^ Department of Obstetrics and Gynecology, Spencer Fox Eccles School of Medicine University of Utah Salt Lake City Utah USA; ^4^ Lowell Center for Sustainable Production University of Massachusetts Lowell Lowell Massachusetts USA; ^5^ Department of Internal Medicine University of Utah Salt Lake City Utah USA; ^6^ Utah Cancer Registry University of Utah Salt Lake City Utah USA; ^7^ Channing Division of Network Medicine, Department of Medicine Brigham and Women's Hospital and Harvard Medical School Boston Massachusetts USA; ^8^ Slone Epidemiology Center, Boston University Chobanian and Avedisian School of Medicine Boston Massachusetts USA

**Keywords:** epidemiology, leukemia, lymphoid neoplasms, lymphoma, myeloid neoplasms, tattoos

## Abstract

**Background:**

Approximately one‐third of US adults have a tattoo, and the prevalence is increasing. Tattooing can result in long‐term exposure to carcinogens and inflammatory and immune responses.

**Methods:**

We examined tattooing and risk of hematologic cancers in a population‐based case–control study with 820 cases diagnosed 2019–2021 and 8200 frequency‐matched controls, ages 18–79 years. We calculated odds ratios (OR) and 95% confidence intervals (CI) using multivariable‐adjusted logistic regression models.

**Results:**

The prevalence of tattooing was 22% among Hodgkin lymphoma (HL) cases, 11% among non‐Hodgkin lymphoma (NHL) cases, 16% among myeloid neoplasm cases, and 15% among controls. Though there were no clear patterns of associations between ever receiving a tattoo and risk of HL, NHL, or myeloid neoplasms overall, in analyses restricted to ages 20–60 years, ever receiving a tattoo (OR 2.06 [95% CI 1.01, 4.20]) and receiving a tattoo 10+ years prior (OR 2.64 [95% CI 1.23, 5.68]) were associated with an aggregated group of rarer mature B‐cell NHLs. We also observed elevated risks for a 10+ year latency for myelodysplastic syndromes and chronic myeloid leukemia (OR 1.48 [95% CI 0.40, 5.41], and OR 1.24 [95% CI 0.45, 3.43], respectively).

**Conclusions:**

Though estimates were imprecise, we found some suggestive evidence that tattooing may be associated with an increased risk of certain hematologic cancer subtypes. With an estimated 46% prevalence of tattooing in US individuals ages 30–49, additional studies are needed to understand the degree to which these exposures may be associated with hematologic cancer risk.

## 1 Introduction

Tattooing has increased over the past two decades with nearly one‐third of adults in the United States estimated to have a tattoo in 2023 [1, 2, 3, 4]. Despite the high prevalence, the long‐term health effects of tattooing are unknown. Commercially available tattoo inks can contain variable levels of heavy metals [5] and other components classified as carcinogenic or probably carcinogenic by the International Agency for Research on Cancer (Groups 1 and 2A), including several polycyclic aromatic hydrocarbons (PAHs) and primary aromatic amines (PAAs) [5, 6, 7, 8]. This includes the PAH benzo(a)pyrene [9] which can alter myeloid and lymphoid cells [10]. In vitro experiments have shown that tattoo inks interact with solar radiation to produce additional toxic compounds and deleterious singlet oxygen [6, 11], a reactive oxygen species which can damage nucleic acids, lipids, and proteins in the skin [12]. Though safety concerns led the European Union to begin regulating tattoo inks in 2022 [13], inks remain unregulated in the United States. After tattoos are placed, inks and their carcinogenic components can travel from the skin through the lymphatic system and accumulate in lymph nodes and other organs [14, 15, 16, 17]. Tattooing may also produce a range of short‐ and long‐term inflammatory and immune responses in the skin [18, 19] and systemically, including sarcoidosis (non‐necrotizing granuloma formation) [20, 21, 22, 23, 24, 25, 26], which is associated with hematologic cancer risk [27, 28, 29].

Taken together, the carcinogenic exposure and inflammatory response from tattooing could plausibly influence the risk of developing hematologic cancers, which include the lymphoid neoplasms Hodgkin lymphoma (HL) and non‐Hodgkin lymphoma (NHL), and the myeloid neoplasms acute myeloid leukemia (AML), myelodysplastic syndromes (MDS), and chronic myeloid leukemia (CML). Indeed, both tattooing and NHL incidence have increased over the past few decades in the majority of western countries, though the causes of the increase in NHL are unknown [30]. As these cancer types are rare, obtaining adequate sample sizes for studying each subtype is difficult. NHL comprises > 60 diverse histologic types, with evidence for both heterogeneity and homogeneity of risk factor associations across subtypes [31, 32]. Risk factors for NHL include genetic factors, adult height, obesity, infections such as Epstein–Barr Virus (EBV), *Helicobacter pylori*, human immunodeficiency virus (HIV)/acquired immunodeficiency syndrome (AIDS), hepatitis B and hepatitis C, certain occupational chemical exposures, organ transplant, and autoimmune diseases including rheumatoid arthritis, Sjogren's syndrome, and celiac disease, as well as subclinical immune dysregulation [33, 34, 35, 36, 37, 38]. Occupational exposures to organic solvents, including benzene, are particularly associated with an increased risk of B‐cell NHL [39]. Exposure to hair dyes has also been linked to an increased risk of non‐Hodgkin lymphoma particularly for the subtypes follicular lymphoma (FL) and chronic lymphocytic leukemia/small lymphocytic leukemia (CLL/SLL) [40]. These associations were strongest among women exposed to hair dyes produced prior to 1980 before many dyes were reformulated to remove mutagenic and carcinogenic components [40]. HL risk factors include family history, poor host control of EBV, HIV/AIDS, and autoimmune and inflammatory conditions, including rheumatoid arthritis [41]. Risk factors for myeloid neoplasms in adults also vary by subtype and include increasing age, therapy with alkylating agents, anthracyclines, topoisomerase II inhibitors, and ionizing radiation [42, 43, 44, 45], exposure to benzene [45, 46, 47] and tobacco smoking [48, 49, 50].

The epidemiologic literature on tattooing and hematologic cancers is sparse. A study in British Columbia, Canada reported no evidence of an association between tattoos and NHL or multiple myeloma overall [51]. The strongest association was an odds ratio (OR) of 1.47 [95% confidence interval (CI) 0.49, 3.66] for T‐cell lymphomas, followed by an OR of 1.27 [95% CI 0.68, 2.30] for an aggregated group of other (e.g., relatively rare) B‐cell NHL subtypes. As this study was conducted between 2000 and 2004, tattoo prevalence in the study population was low (~6%) [51]. A more recent study in Sweden of adults 20–60 years old reported a higher risk of overall lymphoma (incidence rate ratio [IRR] 1.21 [95% CI 0.99, 1.48]), associated with ever receiving a tattoo, with the strongest risk observed for diffuse large B‐cell lymphoma (DLBCL) (IRR 1.30 [95% CI 0.99, 1.71]), and FL (IRR 1.29 [95% CI 0.92, 1.82]) [52]. This study also reported that risk differed by time since first tattoo, as receiving a first tattoo less than 2 years prior was associated with increased risk of overall lymphoma (IRR 1.81 [95% CI 1.03, 3.20]), while receiving a first tattoo at least 11 years prior was suggestively associated with increased risk of overall lymphoma (IRR 1.19 [95% CI 0.94, 1.50]) [52]. There is currently no published research examining tattooing and risk of myeloid neoplasms. Herein, we examine associations of detailed tattoo exposures with risk of HL, NHL, and myeloid neoplasms, as well as their subtypes, in a large general population in the US state of Utah. We are particularly interested in exploring B‐cell NHLs because of the known associations between exposure to solvents and these subtypes and are further interested in assessing associations with tattoos received at least 10 years prior as associations between some exposures and hematologic malignancies have at least a 10‐year latency [53, 54].

## 2 Methods

### 2.1 Study Population

We conducted a population‐based case–control study with incident cancer cases identified by the Utah Cancer Registry and controls selected from the Behavioral Risk Factor Surveillance System (BRFSS) survey [55]. The Utah Cancer Registry meets standards for complete case ascertainment and follow‐up set by the Centers for Disease Control and Prevention's National Program of Cancer Registries (NPCR) and the National Cancer Institute's Surveillance, Epidemiology, and End Results (SEER) program. The BRFSS is an annual population‐based telephone survey of health behaviors administered in all US states. States are able to add questions to their surveys each year. We worked with the Utah Department of Health and Human Services to add three tattoo questions to the 2020 and 2021 Utah BRFSS: (1) What is the total number of tattooing sessions you have had? (2) How many of your tattoos are bigger than your palm? And (3) how old were you when you got your first tattoo? The telephone interviews administered to cases consisted of a shortened version of the BRFSS survey. Cases were asked about the time period 1 year prior to diagnosis in order to collect data about their exposures before they might have changed due to the development of the cancer.

We invited all individuals ages 19–79 years with an incident hematologic cancer diagnosed in Utah between July 1, 2019 and December 31, 2021 to participate. We used ICD‐O‐3 codes to classify lymphoid cases into HL, NHL, and NHL subtypes including CLL/ SLL, DLBCL, FL, and other mature B‐cell NHL; and myeloid cases into AML, MDS, and CML subtypes (Table 1). Cases were invited to participate via a mailed consent cover letter. Individuals were contacted by telephone by a trained interviewer, who reviewed the cover letter with the potential participant, answered any questions, and informed them that by completing the interview they were consenting to participate in the study. As some exposures may have changed prior to diagnosis due to the disease, cases were given a reference date 1 year prior to their diagnosis and asked to think back to that time while answering questions (the reference date for controls was the date of their interview). To ensure comparability to BRFSS controls, cases were considered ineligible if they did not live in Utah 1 year prior to diagnosis, or if they did not live in a private residence or college housing. A total of 1830 cases were eligible for the study; of those, 314 (17%) were found to be deceased prior to contact, 385 (21%) were unable to be reached, and 309 (17%) refused to participate. Surveys were completed by 822 individuals for a response proportion of 45%. We excluded one individual who we later learned did not live in Utah, and one individual who was missing tattoo data, leaving 820 participants (79 HL, 562 NHL, 179 myeloid neoplasms) for analysis. For data collection and management, we utilized REDCap hosted at the University of Utah [56, 57]. The University of Utah Institutional Review Board (IRB) determined this research exempt (#00123466). Participants were given a consent cover letter and were informed that by completing the interview they were consenting to participate in the study.

**TABLE 1:** Hematologic cancer subtype categories and corresponding ICD‐O‐3 codes.

Cancer subtype	ICD‐O‐3 histology codes
Lymphoid	
Hodgkin lymphoma (HL)	
Classical	9650–9655, 9661–9667
Non‐classical	9659
Non‐Hodgkin lymphoma	
Mature B‐cell NHL	
Chronic lymphocytic leukemia/small lymphocytic leukemia (CLL/SLL)	9670, 9823
Diffuse large B‐cell (DLBCL)	9678–9680, 9684 (B), 9688, 9712, 9735, 9737, 9738
Follicular lymphoma (FL)	9690, 9691, 9695, 9698
Other mature B‐cell NHL	
Burkitt lymphoma	9687
Hairy cell leukemia	9940
Lymphoplasmacytic lymphoma/Waldenström macroglobulinemia	9671, 9761
Mantle cell lymphoma	9673
Marginal zone lymphoma	9689, 9699, 9764
Other (T‐cell, pre‐B, pre‐T, or other/not otherwise specified) NHL	9590, 9591, 9597, 9675, 9684 (T/U), 9700–9702, 9705, 9708, 9709, 9714, 9716–9719, 9724–9729, 9811–9818, 9820, 9827, 9831, 9835, 9837, 9948, 9970
Myeloid	
Acute myeloid leukemia (AML)	9840, 9861, 9865–9867, 9869, 9871–9874, 9877–9879, 9891, 9895–9897, 9898, 9910–9912, 9920
Myelodysplastic syndrome (MDS)	9980, 9982–9987, 9989
Chronic myeloid leukemia (CML)	9863, 9875, 9876, 9945, 9946
Other myeloid neoplasm	9808, 9860, 9930, 9950, 9961, 9962, 9975

In total, 21,542 individuals completed the 2020–2021 Utah BRFSS surveys. The response proportions were similar to those in cases, 55% in 2020 and 47% in 2021. We excluded 367 individuals with an unknown age; 1805 individuals over age 78 (to correspond with the maximum age of 79 among cases as cases were asked about the period one year prior to diagnosis); 88 individuals who reported prior hematologic cancer; and 2524 individuals missing tattoo data. In total, 16,758 individuals were eligible for control selection. Controls were frequency matched to cases in a 10:1 ratio on 5‐year age groups, sex (male, female), and race and ethnicity (Hispanic, non‐Hispanic (NH) American Indian or Alaska Native, NH Asian, NH Black, NH Pacific Islander, NH White, NH multiracial, NH other, unknown), as both tattoo prevalence [2] and hematologic cancer risk vary across these demographic factors [58, 59]. A total of 8200 controls were frequency matched to the 820 cases.

### 2.2 Statistical Analysis

We computed counts and proportions of demographic characteristics among lymphoid and myeloid cases and controls. Because we included a subset of BRFSS respondents who were frequency matched to controls rather than the entire BRFSS sample, we did not incorporate the BRFSS survey weights into these analyses. We fit polytomous logistic regression models to compute ORs and 95% CIs associating tattooing variables (ever tattooed; time since first tattoo (< 10 years/10+ years); number of tattoo sessions; number of large tattoos; age at first tattoo) with overall HL, NHL, and myeloid neoplasms, adjusted for sex, age, race and ethnicity (NH White, Hispanic, all other racial and ethnic groups), education level (high school diploma or less vs. some college or more), past cancer diagnosis (yes/no), and ever smoking (yes/no). Race and ethnicity were included as adjustment variables as both hematologic cancer incidence [60] and tattooing prevalence vary by race and ethnicity [2]. We then fit separate polytomous models for the lymphoid subtypes (HL, CLL/SLL, DLBCL, HL, and other mature B‐cell NHL) and the myeloid subtypes (AML, MDS, and CML). To focus on age groups with the highest tattooing prevalence, and to be able to compare our findings with those from the recent Swedish study [52], we performed analyses restricted to individuals ages 20–60 years. Because the risks of these cancers are very low under age 40 (with the exception of HL), in sensitivity analyses we further fit models restricted to individuals diagnosed at ages 40 and older. We also fit models stratified by sex. For sex‐stratified models, race and ethnicity were collapsed to NH White and all other racial and ethnic groups due to small cell sizes.

Because we could not collect data on some potentially relevant confounders (in particular, occupation [39, 61]), we estimated the minimum strength of association that an unmeasured confounder would need to have with both tattooing exposures and cancer risks in order to fully explain the observed ORs, known as an e‐value [62, 63]. All analyses were conducted using R Statistical Software (v4.3.1; R core team 2023; Vienna, Austria).

## 3 Results

A total of 820 cases (641 lymphoid [79 HL, 125 CLL/SLL, 161 DLBCL, 105 FL, 100 other mature B‐cell, 71 other NHL] and 179 myeloid [75 AML, 36 MDS, 47 CML, 21 other myeloid]) and 8200 controls were included in analyses. The majority of cases and controls were ages 60 or older (56% of lymphoid, 60% of myeloid, and 56% of controls), male (56% of lymphoid, 54% of myeloid, and 53% of controls), and NH White (90% of lymphoid, 93% of myeloid, and 90% of controls) (Table 2). Both lymphoid (79%) and myeloid (83%) cases were more likely to have a post‐secondary education than controls (75%). Myeloid cases were more likely to have reported ever smoking (31%) than controls (26%).

**TABLE 2:** Demographics of lymphoid and myeloid neoplasm cases and frequency‐matched controls.

	Controls	Cases	
		Lymphoid	Myeloid
	(*n* = 8200)	(*n* = 641)	(*n* = 179)
	*n* (%)	*n* (%)	*n* (%)
Age			
< 40	1288 (16)	95 (15)	28 (16)
40–49	879 (11)	71 (11)	15 (8)
50–59	1450 (18)	116 (18)	29 (16)
60–69	2627 (32)	202 (32)	64 (36)
70+	1956 (24)	157 (24)	43 (24)
Sex			
Female	3860 (47)	280 (44)	83 (46)
Male	4340 (53)	361 (56)	96 (54)
Race and ethnicity			
Hispanic	510 (6)	43 (7)	*(4)
NH American Indian/Alaskan Native	*(0)	*(0)	0 (0)
NH Asian	60 (1)	*(1)	*(1)
NH black	30 (0)	*(0)	0 (0)
NH Pacific islander	20 (0)	*(0)	0 (0)
NH white	7410 (90)	575 (90)	166 (93)
NH multiracial	100 (1)	*(2)	0 (0)
NH other	*(0)	*(0)	0 (0)
Missing	50 (0.6)	1 (0.2)	4 (2.2)
Education			
Less than high school/High school diploma	2029 (25)	132 (21)	30 (17)
Some college/college graduate or more	6162 (75)	509 (79)	149 (83)
Missing	9 (0.1)	0 (0)	0 (0)
Ever smoking			
Yes	2101 (26)	151 (24)	56 (31)
No	6065 (74)	488 (76)	122 (68)
Missing	34 (0.4)	2 (0.3)	1 (0.6)
History of cancer			
Yes	796 (10)	93 (15)	24 (13)
No	7389 (90)	546 (85)	109 (61)
Missing	15 (0.2)	2 (0.3)	46 (25.7)

Abbreviation: NH, non‐Hispanic.

* Censored due to cell values < 11.

### 3.1 Lymphoid Neoplasms

The prevalence of tattooing was 22% among HL cases, 11% among NHL cases (ranging from 8% to 14% among NHL subtypes), and 15% among controls. Eight percent of both HL and NHL cases and 10% of controls received a first tattoo at least 10 years prior to the reference date. After adjustment for the matching factors and other covariates, ORs for participants who were ever tattooed were less than one for risk of HL (OR 0.66 [95% CI 0.36, 1.21]), overall NHL (OR 0.83 [95% CI 0.61, 1.11]), and for risk of all subtypes of NHL (ORs ranging from 0.65 to 0.81) except the aggregated group of other (e.g., relatively rare) mature B‐cell NHL subtypes (OR 1.13 [95% CI 0.60, 2.10]) (Table 3). Receiving a tattoo at least 10 years prior to the reference date was also associated with decreased HL risk (OR 0.42 [95% CI 0.17, 1.06]) compared with never tattooing. For the other mature B‐cell NHL category, several other tattoo features were also associated with ORs greater than one, compared with never having received a tattoo, albeit based on small numbers of exposed participants. These included: receiving a first tattoo at least 10 years prior to the reference date (OR 1.29 [95% CI 0.66, 2.50]); having four or more tattoo sessions (OR 1.30 [95% CI 0.49, 3.46]); and increasing number of large tattoos (1–2 large tattoos: OR 1.47 [95% CI 0.61, 3.59]; and 3 or more: OR 1.54 [95% CI 0.45, 5.27]). Associations with the other mature B‐cell NHL category were similar for individuals who received their first tattoo before age 20 (OR 1.22 [95% CI 0.46, 3.28]) and those who received their first tattoo at age 20 or after (OR 1.12 [95% CI 0.54, 2.31]). No clear patterns emerged for the other tattoo features in relation to risk of HL or other NHL subtypes.

**TABLE 3:** Associations between tattooing variables and HL and NHL overall and by subtype among individuals ages 19–79 years.

	HL	Overall NHL	NHL subtypes			
			CLL/SLL	DLBCL	FL	Other mature B‐cell
	(*n* = 79)	(*n* = 562)	(*n* = 125)	(*n* = 161)	(*n* = 105)	(*n* = 100)
	OR (95% CI)	OR (95% CI)	OR (95% CI)	OR (95% CI)	OR (95% CI)	OR (95% CI)
Ever tattooed						
No	Ref.	Ref.	Ref.	Ref.	Ref.	Ref.
Yes	0.66 (0.36, 1.21)	0.83 (0.61, 1.11)	0.81 (0.40, 1.67)	0.65 (0.36, 1.17)	0.68 (0.36, 1.31)	1.13 (0.60, 2.10)
Time since first tattoo						
Never tattooed	Ref.	Ref.	Ref.	Ref.	Ref.	Ref
< 10 years	0.94 (0.46, 1.94)	0.76 (0.42, 1.36)	0.46 (0.06, 3.32)	0.78 (0.28, 2.22)	0.95 (0.29, 3.02)	0.70 (0.16, 3.01)
10+ years	0.42 (0.17, 1.06)	0.87 (0.62, 1.21)	0.91 (0.43, 1.95)	0.69 (0.36, 1.33)	0.73 (0.36, 1.48)	1.29 (0.66, 2.50)
Number of tattoo sessions						
Never tattooed	Ref.	Ref.	Ref.	Ref.	Ref.	Ref.
1	0.99 (0.41, 2.38)	0.99 (0.66, 1.48)	0.40 (0.10, 1.64)	1.34 (0.71, 2.53)	0.82 (0.33, 2.07)	1.10 (0.44, 2.77)
2–3	0.50 (0.17, 1.45)	0.65 (0.38, 1.12)	1.17 (0.41, 3.29)	0.14 (0.02, 1.05)	0.81 (0.32, 2.09)	1.02 (0.36, 2.89)
4 or more	0.57 (0.25, 1.35)	0.79 (0.47, 1.33)	1.14 (0.34, 3.77)	0.44 (0.14, 1.44)	0.60 (0.18, 1.99)	1.30 (0.49, 3.46)
Number of large tattoos						
Never tattooed	Ref.	Ref.	Ref.	Ref.	Ref.	Ref.
0	0.33 (0.10, 1.08)	0.80 (0.55, 1.19)	0.30 (0.07, 1.23)	0.91 (0.47, 1.76)	0.77 (0.35, 1.73)	0.81 (0.32, 2.06)
1–2	1.10 (0.54, 2.26)	0.92 (0.58, 1.47)	1.95 (0.81, 4.70)	0.57 (0.20, 1.59)	0.73 (0.26, 2.08)	1.47 (0.61, 3.59)
3 or more	0.48 (0.14, 1.62)	0.70 (0.34, 1.46)	0.77 (0.10, 5.77)	0.27 (0.04, 2.02)	0.75 (0.18, 3.20)	1.54 (0.45, 5.27)
Age at first tattoo						
Never tattooed	Ref.	Ref.	Ref.	Ref.	Ref.	Ref.
< 20 years	0.76 (0.37, 1.57)	0.60 (0.34, 1.05)	0.71 (0.17, 3.02)	0.13 (0.02, 0.94)	0.40 (0.09, 1.71)	1.22 (0.46, 3.28)
20+ years	0.56 (0.23, 1.35)	0.95 (0.69, 1.32)	0.85 (0.39, 1.88)	1.03 (0.58, 1.83)	0.92 (0.47, 1.78)	1.12 (0.54, 2.31)

Abbreviations: CLL, chronic lymphocytic leukemia; DLBCL, diffuse large B‐cell lymphoma; FL, follicular lymphoma; HL, Hodgkin lymphoma; NHL, non‐Hodgkin lymphoma; SLL, small lymphocytic lymphoma.

Analyses restricted to ages 20–60 years old showed similar patterns for HL, DLBCL, and FL to those in the models which included all ages (Table 4). While no increased risk of overall NHL was observed among this age group (OR 1.03 [95% CI 0.71, 1.49]), we observed a suggestive increased risk of CLL/SLL (OR 1.47 [0.60, 3.61]), and a stronger increased risk of the aggregate group of other mature B‐cell NHL (OR 2.06 [95% CI 1.01, 4.20]). The risk of other mature B‐cell NHL was strongest for first tattoos received 10 or more years prior to the reference date (OR 2.64 [1.23, 5.68]), with no association for tattoos received less than 10 years prior.

**TABLE 4:** Associations between tattooing variables and HL and NHL overall and by subtype among individuals ages 20–60 years.

	HL	Overall NHL	NHL subtypes			
			CLL/SLL	DLBCL	FL	Other mature B‐cell
	(*n* = 63)	(*n* = 227)	(*n* = 37)	(*n* = 58)	(*n* = 55)	(*n* = 46)
	OR (95% CI)	OR (95% CI)	OR (95% CI)	OR (95% CI)	OR (95% CI)	OR (95% CI)
Ever tattooed						
No	Ref.	Ref.	Ref.	Ref.	Ref.	Ref.
Yes	0.70 (0.36, 1.34)	1.03 (0.71, 1.49)	1.47 (0.60, 3.61)	0.67 (0.30, 1.51)	0.67 (0.30, 1.49)	2.06 (1.01, 4.20)
Time since first tattoo						
Never tattooed	Ref.	Ref.	Ref.	Ref.	Ref.	Ref.
< 10 years	1.08 (0.49, 2.37)	0.73 (0.36, 1.47)	0.77 (0.10, 5.86)	0.59 (0.14, 2.51)	0.91 (0.27, 3.06)	1.00 (0.22, 4.50)
10+ years	0.45 (0.18, 1.14)	1.20 (0.79, 1.82)	1.80 (0.68, 4.75)	0.88 (0.37, 2.11)	0.60 (0.23, 1.52)	2.64 (1.23, 5.68)
Number of tattoo sessions						
Never tattooed	Ref.	Ref.	Ref.	Ref.	Ref.	Ref.
1	1.01 (0.39, 2.67)	1.20 (0.68, 2.09)	0.62 (0.08, 4.63)	1.72 (0.71, 4.18)	0.78 (0.24, 2.61)	2.53 (0.94, 6.80)
2–3	0.45 (0.13, 1.51)	0.87 (0.47, 1.61)	1.94 (0.56, 6.75)	0.26 (0.03, 1.92)	0.58 (0.17, 1.99)	1.86 (0.62, 5.62)
4 or more	0.69 (0.29, 1.64)	1.03 (0.59, 1.79)	1.92 (0.54, 6.84)	0.43 (0.10, 1.88)	0.66 (0.19, 2.29)	1.90 (0.68, 5.34)
Number of large tattoos						
Never tattooed	Ref.	Ref.	Ref.	Ref.	Ref.	Ref.
0	0.40 (0.12, 1.32)	0.95 (0.56, 1.61)	0.44 (0.06, 3.36)	1.04 (0.40, 2.72)	0.62 (0.21, 1.81)	1.86 (0.69, 5.02)
1–2	1.07 (0.49, 2.35)	1.29 (0.78, 2.12)	3.37 (1.30, 8.74)	0.89 (0.30, 2.60)	0.64 (0.19, 2.18)	2.32 (0.90, 5.99)
3 or more	0.55 (0.16, 1.91)	0.71 (0.30, 1.66)	—	—	0.87 (0.20, 3.86)	2.16 (0.60, 7.77)
Age at first tattoo						
Never tattooed	Ref.	Ref.	Ref.	Ref.	Ref.	Ref.
< 20 years	0.80 (0.36, 1.79)	0.67 (0.34, 1.32)	0.75 (0.09, 5.93)	—	0.23 (0.03, 1.74)	2.03 (0.69, 5.92)
20+ years	0.61 (0.25, 1.49)	1.23 (0.82, 1.85)	1.73 (0.68, 4.40)	1.25 (0.58, 2.67)	0.87 (0.39, 1.96)	2.16 (0.97, 4.78)

Abbreviations: CLL, chronic lymphocytic leukemia; DLBCL, diffuse large B‐cell lymphoma; FL, follicular lymphoma; HL, Hodgkin lymphoma; NHL, non‐Hodgkin lymphoma; SLL, small lymphocytic lymphoma.

Analyses restricted to participants ages 40 and older included approximately 85% of the study sample and showed similar patterns to the overall analyses for HL, overall NHL and the analyzed NHL subtypes for each tattoo exposure variable (Table S1). Sex‐stratified analyses suggested that the associations with tattooing variables appeared to be stronger in males, although data were sparse (Table S2).

### 3.2 Myeloid Neoplasms

The prevalence of tattooing was 16% among myeloid neoplasm cases overall, 13% for AML, 11% for MDS, 21% for CML, and 15% among controls. Eleven percent of AML, 11% of MDS, 17% of CML, and 10% of controls had a first tattoo at least 10 years prior. Having ever been tattooed was not associated with risk of myeloid neoplasms overall (0.99 [0.58, 1.68]) compared with those who did not have a tattoo, though ORs were slightly elevated for MDS (OR 1.17 [95% CI 0.32, 4.26]) and CML (OR 1.10 [95% CI 0.42 2.89]) and inverse for AML (OR 0.63 [95% CI 0.26,1.51]) (Table S1). Compared with never receiving a tattoo, receiving a first tattoo 10 or more years prior to reference date was slightly associated with risk of myeloid neoplasms overall (OR 1.13 [95% CI 0.64, 1.99]), which was due to associations with MDS (OR 1.48 [95% CI 0.40, 5.41]) and CML (OR 1.24 [95% CI 0.45, 3.43]), but not AML (OR 0.81 [95% CI 0.32, 2.06]). Associations with tattooing appeared to be restricted to those who received a first tattoo before the age of 20, compared with those never tattooed; risks were elevated for myeloid neoplasms overall (OR 1.54 [95% CI 0.76, 3.11]) and all three subtypes (MDS: OR 2.67 [95% CI 0.55, 12.9]; CML: OR 1.17 [95% CI 0.30, 4.62]; and AML: OR 1.26 [95% CI 0.44, 3.64]). Analyses restricted to ages 20–60 had sparse cell counts and were similar to results that included ages 19–79 (Table S2). However, the associations appeared somewhat stronger among individuals ages 40 and older (Table S5). Sparse cell counts for sex‐stratified analyses precluded informative comparisons of female‐ and male‐specific associations (Table S6).

### 3.3 Infections Among Cases

Fewer than 10 cases reported ever having hepatitis B, hepatitis C, or HIV, and only 11% of them were tattooed. We do not have data on these infections among controls.

### 3.4 Influence of Unmeasured Confounders

For our observed association between receiving a first tattoo before age 20 and risk of other mature B‐cell NHL subtypes (OR 1.22 [95% CI 0.46, 3.28]), an unmeasured confounder would have to be associated with both tattooing and risk of other mature B‐cell NHL with ORs of at least 1.74 for each to have explained the observed association [62, 63].

## 4 Discussion

This study builds upon the emerging literature regarding tattooing and hematologic cancer risk. As hematologic cancer subtypes are rare, we had small sample sizes for several groups, which led to imprecise estimates. This is a common limitation of studies of hematologic cancer subtypes, but research is urgently needed to elucidate the etiology of these under‐studied cancers [30]. Almost all of the risk estimates we observed included 95% CIs which overlapped null, and it is possible that any of the observed associations could be due to chance. While caution should be used in interpreting our findings due to small sample sizes, we interpret the calculated point estimates as the closest estimate of the true magnitude of association. The suggestive findings observed in this study are notable in the context of the results of a recent published study in Sweden [52] and the biologic plausibility of associations between tattooing and hematologic cancers.

We observed associations of tattooing exposures and increased risk in the group of other mature B‐cell NHLs. This group was comprised of several rarer subtypes (predominantly marginal zone and mantle cell lymphomas) for which we did not have a large enough sample size for individual subtype‐specific analyses. All of the associations we observed with other mature B‐cell NHLs were stronger among individuals ages 20–60 years; ever receiving a tattoo and receiving a first tattoo 10 or more years prior were both associated with over 2‐fold increased risks. Though numbers were sparse, the elevated risk appeared to be strongest among men.

We did not observe that tattooing was associated with elevated risk of HL or most NHL subtypes; if anything, ever receiving a tattoo and receiving a first tattoo at least 10 years prior were associated with a decreased risk of HL. The long‐term effects of tattooing including the prolonged exposure to carcinogens within inks are largely unknown, but it is unlikely that these inverse associations are evidence of a protective effect of tattooing on hematologic cancer risk.

We observed some associations that support tattooing as a potential risk factor for myeloid neoplasms. Receiving a first tattoo prior to age 20 was associated with a 1.5‐fold increased risk of myeloid neoplasms overall, which was largely due to an over 2.5‐fold increased risk associated with the MDS subtype. These suggestive findings were based on small cell sizes and could be due to chance, but are plausible because of the exposure to carcinogens and inflammatory processes associated with tattooing, and warrant further investigation. As MDS increases the risk of developing AML, it may be worth examining whether tattooing could influence MDS progression to AML, which was beyond the scope of the current study.

Some known risk factors for hematologic cancers appear to have a 10‐ to 20‐year latency. For example, some environmental pollutants had a detectable effect on NHL risk starting at least 11 years after exposure [53]. Therefore, it is reasonable to evaluate a similar latency for tattooing exposures and hematologic cancer risk. In this regard, our findings of suggestively increased risk of some rare mature B‐cell NHL subtypes, overall myeloid neoplasms, MDS, and CML associated with receiving a first tattoo 10 or more years prior are plausible, although sparse counts precluded a robust assessment of shorter term risk. Also plausible are the stronger associations with tattoos received at a younger age and risk of certain mature B‐cell NHL subtypes, MDS, and CML in analyses restricted to individuals diagnosed at age 40 and older, as risk of these cancers is generally lower in younger adults, and individuals under age 40 are less likely to have had a tattoo long enough to reach the relevant latency period.

The first published study of tattooing and hematologic cancer risk was conducted in British Columbia (BC) in 2000–2004. While the authors interpreted their findings for ever receiving a tattoo as null for risk of NHL overall and of the subcategories of FL, DLBCL, other B‐cell NHL, and T‐cell NHL, we noted that the OR for risk of the aggregated group of less common B‐cell NHL subtypes was 1.27 [95% CI 0.68, 2.30] compared to never receiving a tattoo [51]. Our study was consistent with these findings, as we observed an OR of 1.13 [95% CI 0.60, 2.10] for the association of ever having been tattooed with the aggregated group of rarer mature B‐cell NHL subtypes, though we observed a higher prevalence of tattooing in our study (15% among controls). Our findings that longer time since first tattoo and increasing number of large tattoos was associated with increased risk of this group of B‐cell NHLs suggest that it is possible that more robust associations may be observed in larger studies.

The second published study of tattooing and lymphoma risk conducted in Sweden of cases diagnosed in 2007–2017 observed associations between ever receiving a tattoo and increased risk of overall lymphoma, DLBCL, and FL [52]. Our findings that ever receiving a tattoo was associated with ORs of less than one for DLBCL (OR 0.81 [95% CI 0.44, 1.48]) and FL (OR 0.69 [95% CI 0.35, 1.34]) were not consistent with the associations observed in Sweden (though they were similar to the results reported by the BC study). The Swedish study included a higher number of cases compared with our study including for DLBCL (*n* = 392 in Sweden compared with *n* = 161 in our study) and FL (*n* = 252 in Sweden compared with *n* = 105 in our study). Thus, our analyses had less statistical power compared with those in the Swedish study. The most consistent result across the three studies was a suggestively elevated risk of other mature B‐cell lymphoma subtypes associated with ever receiving a tattoo (BC: OR 1.27 [95% CI 0.68, 2.30]; Sweden: IRR 1.19 [95% CI 0.74, 1.89]; Utah: OR 1.13 [95% CI 0.60, 2.10]). A meta‐analysis across the three studies assuming fixed effects and inverse variance weighting produced an OR of 1.20 [95% CI 0.87, 1.64]. When we restricted analyses to ages 20–60 as was done in the Swedish study, we observed the strongest associations for this subtype group with confidence intervals which did not overlap null. A meta‐analysis across both studies within ages 20–60 produced an OR of 1.40 [95% CI 0.95, 2.08]. Though neither we nor the BC study observed evidence of associations between tattooing and DLBCL or FL, the increased risk observed in the Swedish study and the established associations between organic solvents and increased risk of B‐cell NHLs warrant future investigations among larger sample sizes, ideally with sufficient numbers to examine more of the relatively rare B‐cell NHL subtypes separately, to further explore the true relationship between tattooing exposures and the potential for these cancer types.

A major strength of this study was the population‐based design utilizing high‐quality SEER registry data for cases and BRFSS data for controls. Like many case–control studies, one of the main limitations of our study was the 45% response proportion among cases, partly explained by the 17% of cases who were deceased prior to first contact. Even with the reasonably large sample of 820 cases, we had small cell sizes within cancer subtype strata and within tattoo exposure categories leading to imprecise estimates. We were also limited by small sample sizes across most non‐white racial and ethnic groups, which prohibited us from examining risk patterns within specific racial and ethnic strata. Moreover, we used a slightly different time period for exposure assessment between cases and controls. While controls were asked to report their answers to the BRFSS survey in the present, we asked cases to report their answers about the period 1 year prior to diagnosis. The rationale for this design was that removing the time window when cases might have changed behaviors due to their illness allows for more scientific rigor. Another limitation is that we did not have data on other risk factors, particularly occupation. Our estimation that an unmeasured confounder would have to have a fairly strong association with both tattooing and risk of NHL provides some reassurance that our findings are not simply due to unmeasured or unknown confounding, although we cannot rule out some influence of residual confounding. Again, this is a common limitation of many studies of hematologic cancers [64]. We were further limited by the lack of additional tattoo exposure data among controls as we were only able to add three tattoo questions to the BRFSS survey. This precluded us from being able to analyze associations between tattoo colors and cancer risk. As there is heterogeneity in tattoo ink composition, having data on tattoo colors would only allow a rough estimation of likely ink components. A more complete investigation of tattoo ink components and cancer risk would require more detailed information such as the brands and batches of ink used, which few individuals if any would be able to report retrospectively. The increased risks that we observed were within subgroup analyses which involved multiple comparisons, and it cannot be ruled out that these were due to chance. However, these risks are plausible in the context of the results from the recent Swedish study [52] and the carcinogenic exposure and inflammatory responses associated with tattooing.

This study lends support to the hypothesis that tattooing could be associated with risk of one or more B‐cell NHL subtypes and of the myeloid neoplasms MDS and CML, though sample sizes and exposure prevalence were small. Ongoing cohort studies that are collecting data on tattooing exposures will need to have very large sample sizes to be able to report stable estimates of risk of hematologic cancer subtypes, which will take years. Though the associations we observed are imprecise, our study offers one of the first assessments of potential long‐term effects of tattooing. Because of the older age distribution of cases compared with the general population, the prevalence of tattooing among our study sample is lower than that of the general population. It is likely that with the high prevalence of tattooing among younger generations (as high as 46% among adults age 30–49 [2]), any associations between tattooing and hematologic cancer will become clearer as the current population ages.

## Author Contributions

**Rachel D. McCarty:** conceptualization (equal), data curation (equal), formal analysis (lead), funding acquisition (equal), investigation (equal), methodology (equal), writing – original draft (lead). **Britton Trabert:** formal analysis (supporting), methodology (equal), supervision (equal), writing – review and editing (equal). **David Kriebel:** formal analysis (supporting), methodology (equal), supervision (equal), writing – review and editing (equal). **Morgan M. Millar:** conceptualization (supporting), methodology (supporting), supervision (equal), writing – review and editing (equal). **Brenda M. Birmann:** methodology (equal), writing – review and editing (equal). **Laurie Grieshober:** conceptualization (supporting), formal analysis (supporting), investigation (supporting), methodology (supporting), writing – review and editing (equal). **Mollie E. Barnard:** conceptualization (supporting), investigation (supporting), methodology (supporting), writing – review and editing (equal). **Lindsay J. Collin:** conceptualization (supporting), formal analysis (supporting), investigation (supporting), methodology (supporting), writing – review and editing (equal). **Katherine A. Lawson‐Michod:** methodology (supporting), writing – review and editing (supporting). **Brody Gibson:** methodology (supporting), writing – review and editing (supporting). **Jenna Sawatzki:** conceptualization (supporting), investigation (supporting), methodology (supporting). **Marjorie Carter:** conceptualization (equal), data curation (equal), investigation (equal), project administration (lead), resources (equal), supervision (equal), writing – review and editing (equal). **Valerie Yoder:** data curation (equal), resources (equal), software (equal), writing – review and editing (equal). **Jeffrey A. Gilreath:** conceptualization (equal), methodology (equal), writing – review and editing (equal). **Paul J. Shami:** conceptualization (equal), methodology (equal), supervision (equal), writing – review and editing (equal). **Jennifer A. Doherty:** conceptualization (lead), formal analysis (supporting), funding acquisition (lead), investigation (lead), methodology (lead), supervision (lead), writing – review and editing (equal).

## Conflicts of Interest

Rachel D. McCarty was supported in part by the National Center for Advancing Translational Sciences of the NIH under Award Number T32TR004392. Lindsay J. Collin was supported by K99CA277580 from the National Cancer Institute of the National Institutes of Health. The other authors declare no conflicts of interest.

## Supporting information


Data S1.

## Data Availability

The data that support the findings of this study are available on request from the corresponding author. The data are not publicly available due to privacy or ethical restrictions. The data from frequency matched controls used in this study are available from the Utah Department of Health and Human Services. Restrictions apply to the availability of these data.
