# Supplementary material for: Tattoos and Risk of Hematologic Cancer: A Population‐Based Case–Control Study in Utah
Source: Cancer Med. 2024 Oct 23;13(20):e70260. doi: 10.1002/cam4.70260 (PMC11499570; doi:10.1002/cam4.70260)
Supplement: Supplementary file 1 — Data S1. [file CAM4-13-e70260-s001.docx]

| Supplemental Table 1. Associations between tattooing variables and myeloid neoplasms overall and by subtype among individuals ages 19-79 years | | | | |  |
| --- | --- | --- | --- | --- | --- |
|  | **Overall** | **AML** | **MDS** | **CML** |  |
|  | (n=179) | (n=75) | (n=36) | (n=47) |  |
|  | OR (95% CI) | OR (95% CI) | OR (95% CI) | OR (95% CI) |  |
| **Ever tattooed** |  |  |  |  |  |
| No | Ref | Ref | Ref | Ref |  |
| Yes | 0.99 (0.58, 1.68) | 0.63 (0.26, 1.51) | 1.17 (0.32, 4.26) | 1.10 (0.42, 2.89) |  |
| **Time since first tattoo** |  |  |  |  |  |
| Never tattooed | Ref | Ref | Ref | Ref |  |
| <10 years | 0.64 (0.19, 2.12) | 0.32 (0.04, 2.46) | - | 0.77 (0.10, 6.25) |  |
| 10+ years | 1.13 (0.64, 1.99) | 0.81 (0.32, 2.06) | 1.48 (0.40, 5.41) | 1.24 (0.45, 3.43) |  |
| **Number of tattoo sessions** |  |  |  |  |  |
| Never tattooed | Ref | Ref | Ref | Ref |  |
| 1 | 0.71 (0.29, 1.77) | 0.29 (0.04, 2.14) | - | 1.12 (0.26, 4.91) |  |
| 2-3 | 1.45 (0.70, 3.01) | 1.17 (0.39, 3.55) | 1.48 (0.19, 11.9) | 1.87 (0.57, 6.13) |  |
| 4 or more | 0.90 (0.37, 2.18) | 0.53 (0.12, 2.37) | 3.83 (0.77, 19.1) | 0.40 (0.05, 3.28) |  |
| **Number of large tattoos** |  |  |  |  |  |
| Never tattooed | Ref | Ref | Ref | Ref |  |
| 0 | 0.61 (0.26, 1.43) | 0.38 (0.09, 1.62) | - | 1.13 (0.32, 3.95) |  |
| 1-2 | 1.57 (0.77, 3.18) | 0.87 (0.25, 3.05) | 2.95 (0.62, 14.1) | 1.69 (0.51, 5.60) |  |
| 3 or more | 1.14 (0.39, 3.30) | 1.07 (0.24, 4.86) | 3.43 (0.40, 29.5) | - |  |
| **Age at first tattoo** |  |  |  |  |  |
| Never tattooed | Ref | Ref | Ref | Ref |  |
| <20 years | 1.54 (0.76, 3.11) | 1.26 (0.44, 3.64) | 2.67 (0.55, 12.9) | 1.17 (0.30, 4.62) |  |
| 20+ years | 0.74 (0.37, 1.51) | 0.32 (0.08, 1.37) | 0.62 (0.08, 4.75) | 1.12 (0.36, 3.45) |  |
| Abbreviations: acute myeloid leukemia (AML); chronic myeloid leukemia (CML); myelodysplastic syndrome (MDS) | | | | | |
| Note: Dashes (-) indicate ORs that failed to compute due to cell sizes equal to ~0 | | | | | |

| Supplemental Table 2. Associations between tattooing variables and myeloid neoplasms overall and by subtype among individuals ages 20-60 years | | | | |  |
| --- | --- | --- | --- | --- | --- |
|  | **Overall** | **AML** | **MDS** | **CML** |  |
|  | (n=76) | (n=33) | (n=10) | (n=26) |  |
|  | OR (95% CI) | OR (95% CI) | OR (95% CI) | OR (95% CI) |  |
| **Ever tattooed** |  |  |  |  |  |
| No | Ref | Ref | Ref | Ref |  |
| Yes | 0.78 (0.39, 1.56) | 0.66 (0.22, 1.94) | 1.13 (0.19, 6.81) | 0.53 (0.15, 1.85) |  |
| **Time since first tattoo** |  |  |  |  |  |
| Never tattooed | Ref | Ref | Ref | Ref |  |
| <10 years | 0.46 (0.10, 2.03) | 0.43 (0.05, 3.57) | - | 0.59 (0.07, 5.05) |  |
| 10+ years | 0.91 (0.43, 1.91) | 0.81 (0.25, 2.65) | 1.35 (0.22, 8.37) | 0.52 (0.13, 2.12) |  |
| **Number of tattoo sessions** |  |  |  |  |  |
| Never tattooed | Ref | Ref | Ref | Ref |  |
| 1 | 0.50 (0.12, 2.12) | - | - | 0.71 (0.09, 5.67) |  |
| 2-3 | 1.34 (0.57, 3.14) | 1.47 (0.42, 5.23) | 1.81 (0.18, 17.80) | 1.15 (0.28, 4.69) |  |
| 4 or more | 0.51 (0.17, 1.53) | 0.56 (0.11, 2.77) | 1.65 (0.16, 17.20) | - |  |
| **Number of large tattoos** |  |  |  |  |  |
| Never tattooed | Ref | Ref | Ref | Ref |  |
| 0 | 0.61 (0.21, 1.79) | 0.29 (0.04, 2.35) | - | 0.80 (0.17, 3.85) |  |
| 1-2 | 1.01 (0.41, 2.45) | 0.92 (0.23, 3.72) | 3.03 (0.50, 18.20) | 0.63 (0.13, 3.17) |  |
| 3 or more | 0.68 (0.19, 2.38) | 1.05 (0.21, 5.25) | - | - |  |
| **Age at first tattoo** |  |  |  |  |  |
| Never tattooed | Ref | Ref | Ref | Ref |  |
| <20 years | 1.15 (0.49, 2.71) | 1.43 (0.42, 4.83) | 1.83 (0.17, 19.80) | 0.55 (0.10, 2.87) |  |
| 20+ years | 0.53 (0.20, 1.42) | 0.22 (0.03, 1.74) | 0.89 (0.09, 8.42) | 0.54 (0.11, 2.56) |  |
| Abbreviations: acute myeloid leukemia (AML); chronic myeloid leukemia (CML); myelodysplastic syndrome (MDS) | | | | | |
| Note: Dashes (-) indicate ORs that failed to compute due to cell sizes equal to ~0 | | | | | |

| Supplemental Table 3. Associations between tattooing variables and HL and NHL overall and by subtype among individuals age 40 and older | | | | | | | | | |  |
| --- | --- | --- | --- | --- | --- | --- | --- | --- | --- | --- |
|  | **HL** | **Overall NHL** | **NHL subtypes** | | | | | | |  |
|  |  |  | **CLL/SLL** | **DLBCL** | | **FL** | | **Other mature B-cell**^a^ | |  |
|  | (n=32) | (n=514) | (n=120) | (n=144) | | (n=100) | | (n=91) | |  |
|  | OR (95% CI) | OR (95% CI) | OR (95% CI) | OR (95% CI) | | OR (95% CI) | | OR (95% CI) | |  |
| **Ever tattooed** |  |  |  |  | |  | |  | |  |
| No | Ref | Ref | Ref | Ref | | Ref | | Ref | |  |
| Yes | 0.61 (0.20, 1.89) | 0.82 (0.59, 1.14) | 0.68 (0.31, 1.50) | 0.81 (0.44, 1.48) | | 0.69 (0.35, 1.34) | | 1.04 (0.51, 2.11) | |  |
| **Time since first tattoo** |  |  |  |  | |  | |  | |  |
| Never tattooed | Ref | Ref | Ref | Ref | | Ref | | Ref | |  |
| <10 years | 1.97 (0.44, 8.78) | 0.75 (0.36, 1.55) | 0.48 (0.07, 3.51) | 0.70 (0.17, 2.88) | | 1.03 (0.32, 3.35) | | - | |  |
| 10+ years | 0.35 (0.08, 1.60) | 0.86 (0.60, 1.23) | 0.75 (0.32, 1.77) | 0.85 (0.44, 1.64) | | 0.62 (0.29, 1.35) | | 1.31 (0.64, 2.68) | |  |
| **Number of tattoo sessions** |  |  |  |  | |  | |  | |  |
| Never tattooed | Ref | Ref | Ref | Ref | | Ref | | Ref | |  |
| 1 | 1.36 (0.39, 4.79) | 0.99 (0.64, 1.53) | 0.41 (0.10, 1.70) | 1.43 (0.73, 2.80) | | 0.84 (0.33, 2.13) | | 0.76 (0.23, 2.44) | |  |
| 2-3 | - | 0.69 (0.39, 1.22) | 1.25 (0.44, 3.52) | 0.19 (0.03, 1.36) | | 0.68 (0.24, 1.93) | | 1.29 (0.45, 3.69) | |  |
| 4 or more | 0.43 (0.05, 3.42) | 0.70 (0.36, 1.35) | 0.45 (0.06, 3.33) | 0.49 (0.12, 2.03) | | 0.46 (0.11, 1.94) | | 1.22 (0.36, 4.09) | |  |
| **Number of large tattoos** |  |  |  |  | |  | |  | |  |
| Never tattooed | Ref | Ref | Ref | Ref | | Ref | | Ref | |  |
| 0 | 0.33 (0.04, 2.55) | 0.75 (0.49, 1.15) | 0.31 (0.08, 1.27) | 0.95 (0.47, 1.91) | | 0.79 (0.35, 1.76) | | 0.56 (0.17, 1.81) | |  |
| 1-2 | 1.45 (0.40, 5.21) | 1.03 (0.61, 1.73) | 1.50 (0.53, 4.28) | 0.65 (0.20, 2.10) | | 0.61 (0.19, 2.01) | | 2.08 (0.85, 5.11) | |  |
| 3 or more | - | 0.67 (0.27, 1.68) | 0.94 (0.13, 6.98) | 0.47 (0.06, 3.50) | | 0.44 (0.06, 3.24) | | 0.84 (0.11, 6.38) | |  |
| **Age at first tattoo** |  |  |  |  | |  | |  | |  |
| Never tattooed | Ref | Ref | Ref | Ref | | Ref | | Ref | |  |
| <20 years | - | 0.74 (0.39, 1.40) | 0.89 (0.21, 3.77) | 0.23 (0.03, 1.66) | | 0.24 (0.03, 1.80) | | 1.65 (0.56, 4.89) | |  |
| 20+ years | 0.95 (0.31, 2.89) | 0.83 (0.57, 1.20) | 0.64 (0.25, 1.61) | 1.04 (0.56, 1.94) | | 0.86 (0.43, 1.71) | | 0.87 (0.37, 2.07) | |  |
| Abbreviations: chronic lymphocytic leukemia (CLL); diffuse large B-cell lymphoma (DLBCL); follicular lymphoma (FL); Hodgkin lymphoma (HL); non-Hodgkin lymphoma (NHL); small lymphocytic lymphoma (SLL) | | | | | | | | | | |
| Note: Dashes (-) indicate ORs that failed to compute due to cell sizes equal to ~0 | | | | |  | |  | |  |  |

| Supplemental Table 4. Associations between tattooing variables and HL, NHL overall, and other mature B-cell NHL, stratified by sex | | | | | | |  |
| --- | --- | --- | --- | --- | --- | --- | --- |
|  | **HL** | | **Overall NHL** | | **Other mature B-cell NHL**^a^ | |  |
|  | **Female** (n=38) | **Male** (n=41) | **Female** (n=242) | **Male** (n=320) | **Female** (n=40) | **Male** (n=60) |  |
|  | OR (95% CI) | OR (95% CI) | OR (95% CI) | OR (95% CI) | OR (95% CI) | OR (95% CI) |  |
| **Ever tattooed** |  |  |  |  |  |  |  |
| No | Ref | Ref | Ref | Ref | Ref | Ref |  |
| Yes | 0.56 (0.25, 1.28) | 0.75 (0.30, 1.86) | 0.75 (0.48, 1.17) | 0.91 (0.61, 1.36) | 0.54 (0.16, 1.89) | 1.67 (0.80, 3.49) |  |
| **Time since first tattoo** |  |  |  |  |  |  |  |
| Never tattooed | Ref | Ref | Ref | Ref | Ref | Ref |  |
| <10 years | 0.83 (0.34, 2.04) | 0.91 (0.26, 3.27) | 1.26 (0.67, 2.39) | 0.14 (0.02,1.00) | 1.53 (0.35, 6.72) | - |  |
| 10+ years | 0.23 (0.05, 1.10) | 0.66 (0.21, 2.08) | 0.57 (0.32, 1.02) | 1.15 (0.76, 1.73) | 0.24 (0.03, 1.83) | 2.21 (1.05, 4.67) |  |
| **Number of tattoo sessions** |  |  |  |  |  |  |  |
| Never tattooed | Ref | Ref | Ref | Ref | Ref | Ref |  |
| 1 | 1.17 (0.39, 3.50) | 0.79 (0.18, 3.47) | 1.36 (0.80, 2.31) | 0.68 (0.35, 1.31) | 1.01 (0.23, 4.36) | 1.21 (0.36, 4.01) |  |
| 2-3 | 0.31 (0.07, 1.42) | 0.82 (0.18, 3.69) | 0.45 (0.19, 1.05) | 0.90 (0.45, 1.82) | - | 2.09 (0.69, 6.28) |  |
| 4 or more | 0.44 (0.14, 1.40) | 0.68 (0.19, 2.45) | 0.30 (0.09, 0.98) | 1.27 (0.70, 2.29) | 0.65 (0.08, 5.20) | 2.10 (0.67, 6.52) |  |
| **Number of large tattoos** |  |  |  |  |  |  |  |
| Never tattooed | Ref | Ref | Ref | Ref | Ref | Ref |  |
| 0 | 0.29 (0.07, 1.27) | 0.38 (0.05, 2.85) | 0.87 (0.53, 1.43) | 0.71 (0.38, 1.32) | 0.58 (0.13, 2.49) | 1.06 (0.32, 3.55) |  |
| 1-2 | 0.87 (0.32, 2.36) | 1.36 (0.49, 3.80) | 0.61 (0.26, 1.44) | 1.16 (0.66, 2.04) | 0.65 (0.08, 5.19) | 2.07 (0.76, 5.63) |  |
| 3 or more | 0.58 (0.13, 2.70) | 0.32 (0.04, 2.54) | 0.29 (0.04, 2.13) | 0.94 (0.42, 2.11) |  | 2.58 (0.71, 9.35) |  |
| **Age at first tattoo** |  |  |  |  |  |  |  |
| Never tattooed | Ref | Ref | Ref | Ref | Ref | Ref |  |
| <20 years | 0.56 (0.20, 1.53) | 1.03 (0.35, 2.97) | 0.35 (0.11, 1.16) | 0.76 (0.40, 1.45) | - | 1.93 (0.69, 5.39) |  |
| 20+ years | 0.57 (0.19, 1.73) | 0.48 (0.11, 2.10) | 0.89 (0.56, 1.41) | 1.04 (0.65, 1.66) | 0.71 (0.21, 2.41) | 1.61 (0.65, 3.98) |  |
| Abbreviations: Hodgkin lymphoma (HL); non-Hodgkin lymphoma (NHL) | | | | | | |  |
|  |  |  |  |  |  |  |  |

| Supplemental Table 5. Associations between tattooing variables and myeloid neoplasms overall and by subtype among individuals age 40 and older | | | | |  |
| --- | --- | --- | --- | --- | --- |
|  | **Overall** | **AML** | **MDS** | **CML** |  |
|  | (n=151) | (n=62) | (n=34) | (n=36) |  |
|  | OR (95% CI) | OR (95% CI) | OR (95% CI) | OR (95% CI) |  |
| **Ever tattooed** |  |  |  |  |  |
| No | Ref | Ref | Ref | Ref |  |
| Yes | 1.05 (0.57, 1.92) | 0.62 (0.21, 1.84) | 1.34 (0.37, 4.84) | 1.29 (0.44, 3.83) |  |
| **Time since first tattoo** |  |  |  |  |  |
| Never tattooed | Ref | Ref | Ref | Ref |  |
| <10 years | 0.45 (0.06, 3.25) | - | - | - |  |
| 10+ years | 1.21 (0.65, 2.27) | 0.78 (0.26, 2.33) | 1.65 (0.45, 5.99) | 1.63 (0.54, 4.86) |  |
| **Number of tattoo sessions** |  |  |  |  |  |
| Never tattooed | Ref | Ref | Ref | Ref |  |
| 1 | 0.70 (0.25, 1.94) | 0.39 (0.05, 2.86) | - | 0.71 (0.09, 5.46) |  |
| 2-3 | 1.65 (0.73, 3.73) | 1.39 (0.39, 4.87) | 1.62 (0.20, 13.0) | 2.34 (0.62, 8.84) |  |
| 4 or more | 0.92 (0.28, 3.04) | - | 4.70 (0.96, 23.0) | 0.89 (0.11, 7.36) |  |
| **Number of large tattoos** |  |  |  |  |  |
| Never tattooed | Ref | Ref | Ref | Ref |  |
| 0 | 0.64 (0.26, 1.62) | 0.51 (0.12, 2.17) | - | 1.02 (0.23, 4.57) |  |
| 1-2 | 2.17 (0.99, 4.73) | 1.21 (0.27, 5.39) | 3.43 (0.72, 16.4) | 2.30 (0.59, 8.92) |  |
| 3 or more | 0.58 (0.08, 4.29) | - | 4.25 (0.50, 36.1) | - |  |
| **Age at first tattoo** |  |  |  |  |  |
| Never tattooed | Ref | Ref | Ref | Ref |  |
| <20 years | 1.67 (0.68, 4.06) | 1.33 (0.29, 6.12) | 3.15 (0.66, 15.1) | 1.71 (0.34, 8.50) |  |
| 20+ years | 0.83 (0.39, 1.76) | 0.43 (0.10, 1.84) | 0.66 (0.08, 5.08) | 1.16 (0.32, 4.19) |  |
| Abbreviations: acute myeloid leukemia (AML); chronic myeloid leukemia (CML); myelodysplastic syndrome (MDS) | | | | | |

Note: Dashes (-) indicate ORs that failed to compute due to cell sizes equal to ~0

| Supplemental Table 6. Associations between tattooing variables and myeloid neoplasms overall and by subtype, stratified by sex | | | | | | | | |  | |
| --- | --- | --- | --- | --- | --- | --- | --- | --- | --- | --- |
|  | **Overall** | | **AML** | | **MDS** | | **CML** | | | |
|  | **Female** (n=83) | **Male** (n=96) | **Female** (n=40) | **Male** (n=35) | **Female** (n=10) | **Male** (n=26) | **Female** (n=25) | | **Male** (n=22) | |
|  | OR (95% CI) | OR (95% CI) | OR (95% CI) | OR (95% CI) | OR (95% CI) | OR (95% CI) | OR (95% CI) | | OR (95% CI) | |
| **Ever tattooed** |  |  |  |  |  |  |  | |  | |
| No | Ref | Ref | Ref | Ref | ** | Ref | Ref | | Ref | |
| Yes | 1.16 (0.54, 2.49) | 0.82 (0.38, 1.74) | 0.47 (0.12, 1.81) | 0.80 (0.25, 2.56) |  | 1.44 (0.38, 5.41) | 1.69 (0.46, 6.18) | | 0.62 (0.13, 3.02) | |
| **Time since first tattoo** |  |  |  |  |  |  |  | |  | |
| Never tattooed | Ref | Ref | Ref | Ref | ** | Ref | Ref | | Ref | |
| <10 years | 0.72 (0.16, 3.21) | 0.54 (0.07, 4.21) | - | 0.87 (0.10, 7.97) |  | - | 1.21 (0.13, 10.9) | | - | |
| 10+ years | 1.40 (0.61, 3.22) | 0.90 (0.41, 1.99) | 0.75 (0.19, 3.00) | 0.80 (0.22, 2.93) |  | 1.69 (0.44, 6.44) | 1.96 (0.49, 7.92) | | 0.70 (0.14, 3.49) | |
| **Number of tattoo sessions** |  |  |  |  |  |  |  | |  | |
| Never tattooed | Ref | Ref | Ref | Ref | ** | Ref | Ref | | Ref | |
| 1 | 1.07 (0.32, 3.55) | 0.47 (0.11, 1.97) | - | 0.56 (0.07, 4.34) |  | - | 1.34 (0.16, 10.9) | | 0.96 (0.12, 7.65) | |
| 2-3 | 1.82 (0.68, 4.82) | 0.98 (0.29, 3.26) | 1.39 (0.34, 5.61) | 0.82 (0.10, 6.50) |  | 1.74 (0.21, 14.5) | 3.10 (0.65, 14.7) | | 1.11 (0.13, 9.27) | |
| 4 or more | 0.58 (0.13, 2.64) | 1.14 (0.38, 3.43) | - | 1.19 (0.24, 5.93) |  | 4.40 (0.81, 23.9) | 0.81 (0.09, 7.73) | | - | |
| **Number of large tattoos** |  |  |  |  |  |  |  | |  | |
| Never tattooed | Ref | Ref | Ref | Ref | ** | Ref | Ref | | Ref | |
| 0 | 0.41 (0.10, 1.74) | 0.83 (0.29, 2.36) | - | 0.89 (0.19, 4.10) |  | - | 0.81 (0.10, 6.84) | | 1.78 (0.38, 8.46) | |
| 1-2 | 3.18 (1.24, 8.15) | 0.52 (0.12, 2.22) | 1.55 (0.35, 6.87) | - |  | 3.03 (0.61, 15.1) | 5.07 (1.08, 23.9) | | - | |
| 3 or more | 0.87 (0.11, 6.95) | 1.30 (0.37, 4.51) | - | 1.67 (0.34, 8.28) |  | 3.92 (0.42, 36.5) | - | | - | |
| **Age at first tattoo** |  |  |  |  |  |  |  | |  | |
| Never tattooed | Ref | Ref | Ref | Ref | ** | Ref | Ref | | Ref | |
| <20 years | 1.21 (0.36, 4.10) | 1.57 (0.66, 3.72) | 0.80 (0.14, 4.68) | 1.44 (0.38, 5.51) |  | 2.41 (0.49, 11.9) | 0.83 (0.08, 8.71) | | 1.69 (0.33, 8.76) | |
| 20+ years | 1.18 (0.50, 2.77) | 0.33 (0.08, 1.38) | 0.28 (0.04, 2.18) | 0.40 (0.05, 3.13) |  | 0.75 (0.09, 5.93) | 2.31 (0.62, 8.60) | | - | |
| Abbreviations: acute myeloid leukemia (AML); chronic myeloid leukemia (CML); myelodysplastic syndrome (MDS) | | | | | | | |  | |  |
| **Model failed to converge or unable to fit model due to 0 in cell | | | | | | | |  | |  |
